# Two new species of *Neoperla* (Plecoptera, Perlidae) from Dabie Mountains of China

**DOI:** 10.3897/zookeys.438.8230

**Published:** 2014-09-01

**Authors:** Wei-Hai Li, Sheng-Quan Zhang

**Affiliations:** 1 Department of Plant Protection, Henan Institute of Science and Technology, Xinxiang, Henan 453003, China; 2 Administative Bureau of Liankangshan National Nature Reserve, Xin County, Xinyang, Henan 465550, China

**Keywords:** Plecoptera, Perlidae, Neoperla nigromarginata, Neoperla similiflavescens, new species, China

## Abstract

Two new species of the stonefly genus *Neoperla*, *N. nigromarginata* **sp. n.** and *N. similiflavescens* **sp. n.**, are described from Dabie Mountains of Central China in the Liankangshan National Nature Reserve. The new species are compared with related congeners.

## Introduction

*Neoperla* is the most species-rich stonefly genus in the family Perlidae in China (Du et al. 1999, Sivec et al. 1988, DeWalt et al. 2014). To date, seventy-seven *Neoperla* species from China have been reported and the systematic study of this genus in China includes contributions from Chu (1929), Du (1999, 2000a, 2000b), Du (1998), Du and Sivec (2004, 2005), Du and Wang (2005, 2007), Du et al. (1999, 2001), Kong Li et al. (2014), Li et al. (2011a), Li et al. (2011b), Li and Wang (2011), Li et al. (2012a), Li et al. (2012b), Li and Li (2013a, b), Li et al. (2013a), Li et al. (2013b), Li et al. (2014a), Li et al. (2014b), Qin et al. (2013), Sivec and Zwick (1987), Wang et al. (2013a), Wang et al. (2013b), Wu (1935, 1938, 1948, 1962, 1973), Wu and Claassen (1934), Yang and Yang (1990, 1991), and Yang and Yang (1992, 1993, 1995a, 1995b, 1996, 1998).

In the present paper, two new species of *Neoperla*, *Neoperla nigromarginata* sp. n. and *Neoperla similiflavescens* sp. n., are described from Liankangshan National Nature Reserve of China, based on the specimens collected in the recent two years. The Reserve is located in the northern escarpment of Dabie Mountains, the border of Henan and Hubei provinces of Central China. The Reserve also includes the watershed of Yangtze River-Huaihe River basins and the northernmost boundary of subtropical zone (Ye et al. 2002).

## Material and methods

The specimens used in this study were collected by light trap. Types and other examined material are deposited in the Insect Collection of Henan Institute of Science and Technology (HIST), Xinxiang, and the Entomological Museum of China Agricultural University (CAU), Beijing. They were examined with the aid of a Motic SMZ 168 microscope and the color illustrations were captured using digitalized Motic Images Advanced 3.2 software. All specimens were kept in 80% ethanol. Aedeagi were everted using the cold maceration technique of Zwick (1983). Terminology follows that of Sivec et al. (1988). All the scale lines in the figures represent 1.0 mm.

## Results

### Neoperla nigromarginata

Taxon classificationAnimaliaPlecopteraPerlidae

Li & Zhang sp. n.

http://zoobank.org/14EAFB99-1450-4DF2-8ACF-E7FDB99620A3

Figs 1 –2

#### Type material.

Holotype. male (HIST), China: Henan Province, Xinyang City, Xin County, Liankangshan National Nature Reserve, Laomiao Protection station, 31°64,39'N, 114°87,95'E, light trap, 17 June 2014, W.H. Li. Paratypes: 4 females (2 in CAU), the same locality and data as holotype.

#### Adult habitus.

**Figure 1.**
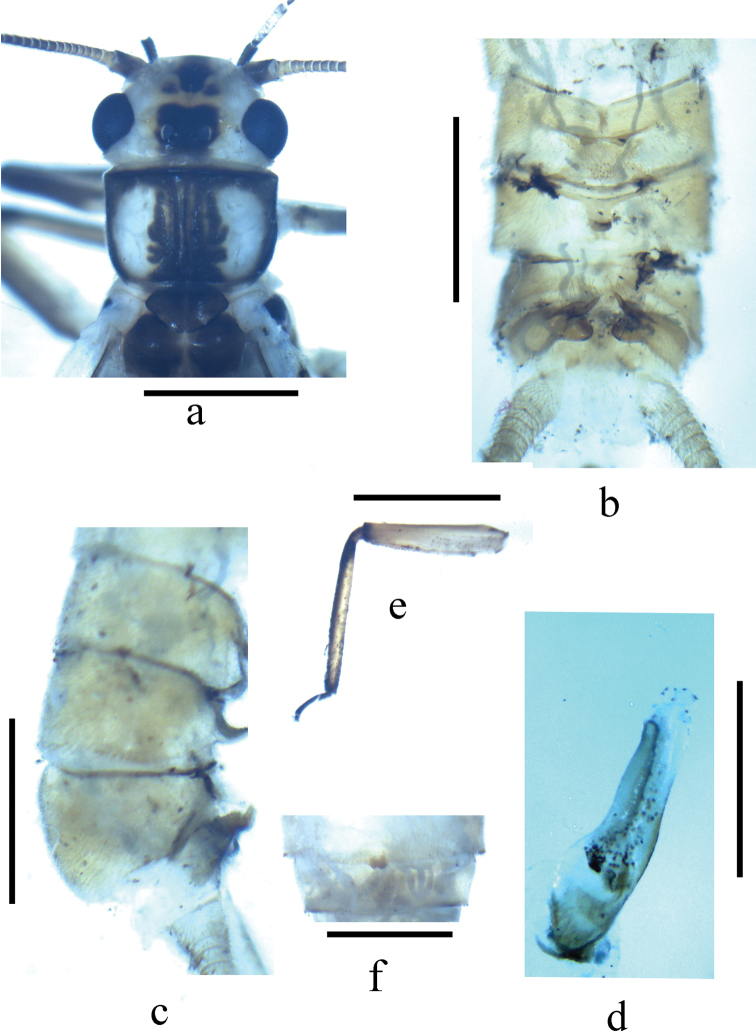
*Neoperla nigromarginata* Li & Zhang, sp. n. Male (**a–e**) **a** Head and pronotum, dorsal view **b** Terminalia, dorsal view **c** Terminalia, lateral view **d** Aedeagus before eversion, lateral view **e** Hindleg **f** Female subgenital fig, ventral view.

Distance between ocelli slightly wider than diameter of the ocellus. Head pale yellow to brownish with black areas, slightly wider than pronotum, with a black rectangular area covering ocelli, a large black anterior spot and two small lateral spots present on frons (Fig. 1a); compound eyes black, antennae dark brown except several basal segments which are brown; maxillary palpi dark brown. Pronotum with black median areas of rugosities and lateral margins around the kidney shaped pale disc area (Fig. 1a); wings subhyaline, veins dark brown; legs dark brown with femora and inner part of tibia brownish to brown (Fig. 1e). Cerci dark brown, basal segments brown.

**Figure 2.**
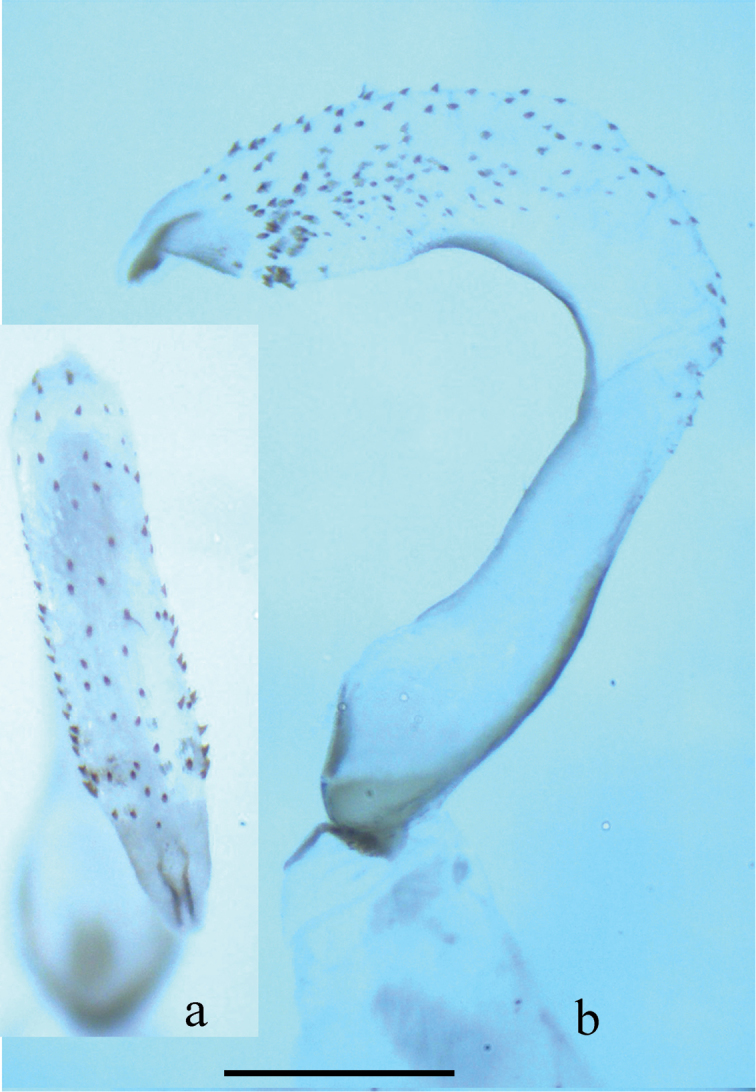
*Neoperla nigromarginata* Li & Zhang, sp. n. Male. **a** Dorsal aspect of aedeagal sac, top view **b** Aedeagus, lateral view. Note that the spines in **b** appear lightly pigmented and unclear, actually they are located on the lower surface of the sac, and are seen from beneath through the cuticle.

Male. Forewing length 11.8 mm. Tergum 7 with an anteromedian pair of sclerotized, upraised, nipple-shaped processes and a distal subquadrate process on posterior margin, covered with small sensilla basiconica (Fig. 1b). Tergum 8 with a recurved tongue shaped process, fringed with small spines at its distal margin (Figs 1b). Tergum 9 without patches of sensilla basiconica. Hemitergal processes of tergum 10 with a swollen wide base medially curved with sharp tip (Fig. 1b). Aedeagal tube nearly straight, with basoventral and dorsal sclerites. Aedeagal sac membranous but basal half heavily sclerotized ventrally (Figs 1d, 2b). Sac about as long as tube and curved ventrally forming a right angle to tube, dorsal surface with 2-3 irregular rows of numerous small spines (Fig. 2a); lateral surface of sac with a large patch of numerous small spines at subapcial region (Fig. 2b); a pair of flagella present more evident in uneverted sac apex in dorsal view (Fig. 2a).

#### Female.

Forewing length 13.4–14.0 mm. General color pattern similar to male. Sternum 7 slightly sclerotized except the posterior margin slightly produced forming sclerotized subgenital fig. Subgenital fig a small dark brown tab with slightly emarginate tip (Fig. 1f). Sternum 8 moderately sclerotized medially, posterior margin slightly produced in a narrow wide lobe.

#### Etymology.

The specific epithet refers to the dark lateral margins of pronotum.

#### Distribution.

China (Henan Province).

#### Diagnosis and remarks.

**Figure 3.**
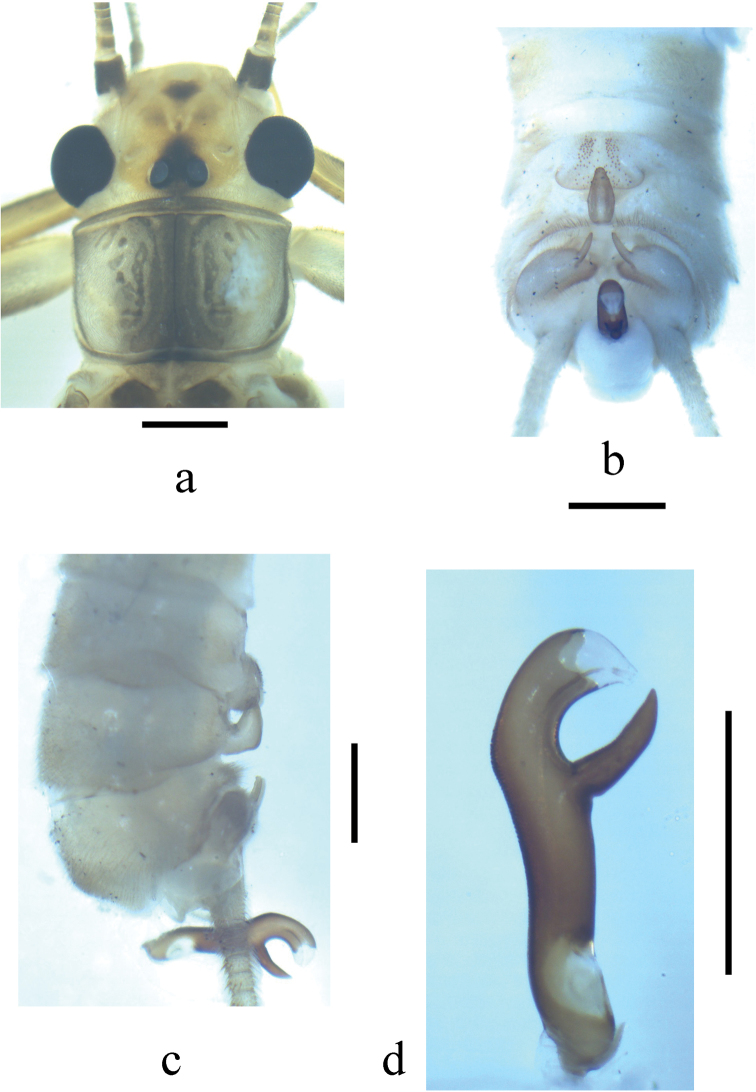
*Neoperla similiflavescens* Li & Zhang, sp. n. Male. **a** Head and pronotum, dorsal view **b** Terminalia, dorsal view **c** Terminalia, lateral view **d** Aedeagus, lateral view.

**Figure 4.**
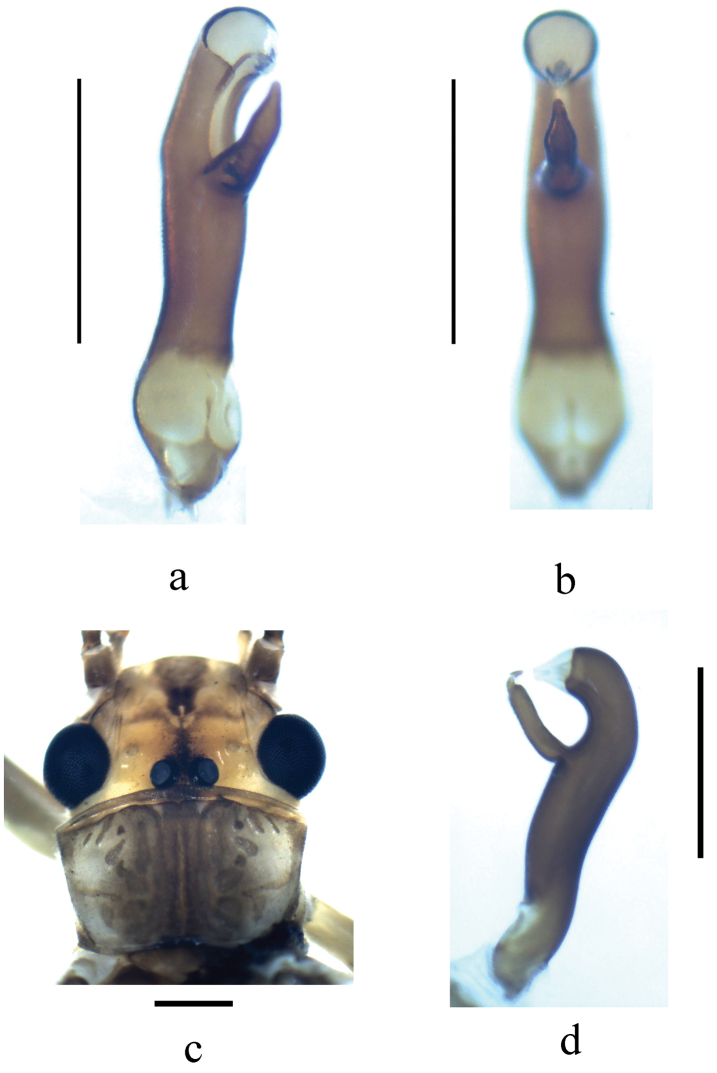
**a–b** *Neoperla similiflavescens* Li & Zhang, sp. n. Male. **c–d** *Neoperla flavescens* Chu. Male. **a** Aedeagus, oblique ventral view **b** Aedeagus, ventral view **c** Head and pronotum, dorsal view **d** Aedeagus, lateral view. *Neoperla flavescens* Chu for comparison. 1 male from Henan Province, Luoyang City, Song County, Cecun town, Muzhaling, 2012.VIII.19, Weihai Li.

The new species is a typical member of the *Neoperla montivaga* species group as defined by Zwick (1983). The new species has similar head pattern, terminalia and paired apical flagella of the aedeagal sac with *Neoperla flagellata* Li & Murányi, 2012 and *Neoperla tuberculata* Wu, 1937 (Figs 1–4, 7 in Li et al. 2012, Figs 5 and 10 in Li et al. 2013). However, *Neoperla nigromarginata* is easily distinguishable from the latter two species by the distinctively pigmented pronotal lateral margins, short aedeagal sac (nearly as long as tube) with only dorsal spine patch at base and absence of a sac loop (Figs 1a, 2). In *Neoperla flagellata* and *Neoperla tuberculata*, both have long aedeagal sac (at least 1.5× as long as tube) forming a loop with lateral spine patches at base of aedeagal sac (Figs 5–9 in Li et al. 2012b, Figs 5 & 11 in Li et al. 2013a).

### Neoperla similiflavescens

Taxon classificationAnimaliaPlecopteraPerlidae

Li & Zhang sp. n.

http://zoobank.org/5071BF60-1E10-4ABF-A529-7D88DC4A71D3

Figs 3 –4

#### Type material.

Holotype. male (HIST), China: Henan Province, Xinyang City, Xin County, Liankangshan National Nature Reserve, Laomiao Protection station, 31°64,39'N, 114°87,95'E, light trap, 15 June 2014, W.H. Li.

#### Male.

Forewing length 13.9 mm. Distance between ocelli barely as wide as diameter of the ocellus. Head slightly wider than pronotum, mostly yellow brown, lateral margins and occiput behind compound eyes pale, a triangular dark area covering ocelli, a dark spot in front of M-line and U-shaped brownish spot between M-line and ocellus, M-line pale; antennae brown to dark brown, scape darker; compound eyes black; mouthparts brown (Fig. 3a). Pronotum brownish with wide darker median stripe and scattered markings of rugosities, legs brown; wings pale brown with dark vein. Abdomen brownish.

*Terminalia*. Tergum 7 with trapezoidal median process at posterior margin, covered by numerous tiny sensilla basiconica, anterior and lateral margins forming elevated figau but with posteromedian concativity in which process of tergum 8 lies. Sclerotized process of tergum 8 recurved backward and triangular in shape. Tergum 9 with two submedial patches of long hairs. Hemitergal processes of tergum 10 strongly sclerotized and straight (Figs 3b and c). Aedeagus heavily sclerotized, subapical ventral projection triangular in lateral view and gradually tapering to a triangular tip (Figs 3d, 4a and b).

#### Female.

Unknown.

#### Etymology.

The specific epithet refers to the similarity to *Neoperla flavescens* Chu, 1929.

#### Distribution.

China (Henan Province).

#### Diagnosis and remarks.

The new species may be assigned to the *Neoperla montivaga* species group as defined by Zwick (1983), because of the incomplete sclerotization of the aedeagal tube in ventral aspect (Fig. 4a). The new species seems closely related to *Neoperla flavescens* Chu originally known from Zhejiang Province, and recently also found from several provinces (Henan, Fujian and Shanxi) of China (Li et al. 2011a, Li et al. 2014). They are similar in general features of male terminalia and the aedeagal tube. However, the new species can be easily separated from *Neoperla flavescens* Chu by the shape of ventral projection of aedeagal tube. The projection in *Neoperla similiflavescens* is generally triangular in lateral view and tapers to a triangular tip (Fig. 3d) whereas it is generally finger-like and abruptly constricted subapically in a nipple-like tip in *Neoperla flavescens* (Figs 4d & 5 in Li et al. 2011a). Their general body color and head pattern also differ to some degree: *Neoperla similiflavescens* is generally brownish in color and spots on frons are well defined and small (Fig. 3a) while in *Neoperla flavescens*, the general body color is brown and darker, the spots on frons are larger and obscure (Fig. 4c, Fig. 1 in Li et al. 2011a, Fig. 1a in Li et al. 2014a).

## Concluding remarks

The present study is based on the insect collection of two years surveys to the Liankangshan National Nature Reserve organized by administrative bureau of the Reserve. In the year 2013 only several stonefly nymphs were collected before the present *Neoperla* finding. Previous study on the stoneflies from the Dabie Mountains only includes the description of *Neoperla jigongshana* Li & Li, 2014 (Li et al. 2014b) from Mountain Jigongshan, about 100 km far from the Reserve. However, there was no record of any group of stoneflies in previous studies on the insect fauna of the Liankangshan Nature Reserve. In this study, two additional new species are described and up to 3 *Neoperla* species are known from the Dabie Mountains presently.

## Supplementary Material

XML Treatment for Neoperla nigromarginata

XML Treatment for Neoperla similiflavescens

## References

[B1] Chu Y-T (1929) Descriptions of four new species and one new genus of stoneflies in the family Perlidae from Hangchow. The China Journal 10: 88–92

[B2] DeWalt RE Maehr, MD Neu-Becker U Stueber G (2014) Plecoptera Species File Online. Version 5.0/5.0. 6/30/2014. http://Plecoptera.SpeciesFile.org

[B3] Du Y-Z (1998) Two new record species of genus *Neoperla* Needham (Plecoptera: Perlidae: Perlinae) from China. Journal of Zhejiang Agricultural University 24: 392–394

[B4] Du Y-Z (1999) Plecoptera. In: Huang BK (Ed) Fauna of Insects in Fujian Province of China.Vol. 3. Fujian Science and Technology Publishing house, Fuzhou, Fujian, 301–335

[B5] Du Y-Z (2000a) *Neoperla magisterchoui*, a new species of the genus *Neoperla* Needham (Plecoptera: Perlidae) from China. In: Zhang YL (Ed) Systematic and Faunistic Research on Chinese Insects.Proceedings of the 5^th^ National Congress of Insect Taxonomy. China Agriculture Press, Beijing, 1–3

[B6] Du Y-Z (2000b) Two new species of the genus *Neoperla* Needham (Plecoptera: Perlidae: Perlinae) from Guizhou, China. Entomotaxonomia 22: 1–5

[B7] Du Y-Z Sivec I (2004) Plecoptera: Perlidae, Nemouridae, Leuctridae. In: Yang XK (Ed) Insects from Mt. Shiwandashan area of Guangxi. China forestry Publishing House, Beijing, 39–45

[B8] Du Y-Z Sivec I (2005) Plecoptera. In: Yang XK (Ed) Insect Fauna of Middle-west Qinling Range and South Mountains of Gansu Province. Science Press, Beijing, 38–54

[B9] Du YZ Sivec I He J-H (1999) A checklist of the Chinese species of the family Perlidae (Plecoptera: Perloidea). Acta Entomologica Slovenica 7: 59–67

[B10] Du Y-Z Sivec I Zhao M-S (2001) Plecoptera. In: Wu H Pan CW (Eds) Insects of Tianmushan National Nature Reserve. Science Press, Beijing, 69–80

[B11] Du Y-Z Wang Z-J (2005) Plecoptera: Leuctridae, Nemouridae, Perlidae and Peltoperlidae. In: Yang MF Jin DC (Eds) Insects from Dashahe Nature Reserve of Guizhou. Guizhou People Press, Guiyang, Guizhou, 51–57

[B12] Du Y-Z Wang Z-J (2007) Nemouridae and Perlidae. In: Li ZZ Yang MF Jin DC (Eds) Insects from Mountain Leigongshan Landscape of Guizhou. Guizhou Science and Technology Publishing house, Guiyang, Guizhou, 84–90

[B13] Kong F-B Lv J-F Li W-H (2014) Three new species of *Neoperla* in the *montivaga* group (Plecoptera: Perlidae) from China. Zootaxa 3841(3): 429–438. doi: 10.11646/zootaxa.3841.3.7 25082049 10.11646/zootaxa.3841.3.7

[B14] Li S Li W-H Wang Y-B (2014a) Female morphology of *Neoperla flavescens* Chu, 1929. Journal of Henan Institute of Science and Technology 42: 28–30

[B15] Li W-H Li X-P (2013a) A new species of the genus *Neoperla* (Plecoptera: Perlidae) from China. Acta Zootaxonomica Sinica 38: 75–77

[B16] Li W-H Li X-P (2013b) A replacement name for *Neoperla siveci* Li and Li (Plecoptera: Perlidae). Illiesia 9: 109

[B17] Li W-H Liang H-Y Li W-L (2013a) Review of *Neoperla* (Plecoptera: Perlidae) from Zhejiang Province, China. Zootaxa 3652(3): 353–369. doi: 10.11646/zootaxa.3652.3.4 10.11646/zootaxa.3652.3.4 26269838

[B18] Li W-H Murányi D Wang R-F (2014a) Species of *Neoperla* (Plecoptera: Perlidae) from Yunnan Province, China. Zootaxa 3753: 1–9. doi: 10.11646/zootaxa.3753.1.1 24872275 10.11646/zootaxa.3753.1.1

[B19] Li W-H Li S Feng G-W Wang Y-B (2014b) Species of *Neoperla* (Plecoptera: Perlidae) from Henan Province, China. Zootaxa 3838(2): 174–182. doi: 10.11646/zootaxa.3838.2.2 25081767 10.11646/zootaxa.3838.2.2

[B20] Li W-H Wang R-F (2011) A new species of *Neoperla* (Plecoptera: Perlidae) from China. Entomological News 122: 261–264. doi: 10.3157/021.122.0308

[B21] Li W-H Wang H-L Lu W-Y (2011a) Species of the genus *Neoperla* (Plecoptera: Perlidae) from Henan, China. Zootaxa 2735: 57–63

[B22] Li W-H Wu L-M Zhang H-R (2011b) A new species of the genus *Neoperla* (Plecoptera: Perlidae) from Henan, China. Acta Zootaxonomica Sinica 36: 33–35

[B23] Li W-H Wang G-Q Lu W-Y (2012a) Species of *Neoperla* (Plecoptera: Perlidae) from Hubei, China. Zootaxa 3478: 32–37

[B24] Li W-H Wang G-Q Li W-L Murányi D (2012b) Review of *Neoperla* (Plecoptera: Perlidae) from Guangdong Province of China. Zootaxa 3597: 15–24

[B25] Li W-H Wang G-Q Qin X-F (2013b) Two new species of *Neoperla* (Plecoptera: Perlidae) from China. ZooKeys 290: 21–30. doi: 10.3897/zookeys.290.456823794856

[B26] Qin X-F Murányi D Wang G-Q Li Q-H (2013) Stoneflies of the genus *Neoperla* (Plecoptera, Perlidae) from Wuyi Mountain National Nature Reserve, Fujian of China. ZooKeys 326: 1–16. doi: 10.3897/zookeys.326.5911 24039532 10.3897/zookeys.326.5911 PMC3763690

[B27] Sivec I Zwick P (1987) Some *Neoperla* (Plecoptera) from Taiwan. Beiträge zur Entomologie 37: 391–405

[B28] Sivec I Stark BP Uchida S (1988) Synopsis of the world genera of Perlinae (Plecoptera: Perlidae). Scopolia 16: 1–66

[B29] Wang H-L Wang G-Q Li W-H (2013a) Two new species in the subfamily Perlinae (Plecoptera, Perlidae) from China. ZooKeys 313: 81–90. doi: 10.3897/zookeys.313.5460 23840166 10.3897/zookeys.313.5460 PMC3701232

[B30] Wang G-Q Li W-H Yang J (2013b) New species and records of the stonefly genus *Neoperla* (Plecoptera, Perlidae) from Jinhuacha Nature Reserve, Guangxi of China. ZooKeys 351: 37–48. doi: 10.3897/zookeys.351.6259 24294087 10.3897/zookeys.351.6259 PMC3837494

[B31] Wu C-F (1935) Aquatic insects of China. Article XXI. New species of stoneflies from East and South China. (Order Plecoptera). Peking Natural History Bulletin 9: 227–243

[B32] Wu C-F (1938) Plecopterorum sinensium: A monograph of stoneflies of China (Order Plecoptera). Yenching University, 225 pp

[B33] Wu C-F (1948) Fourth supplement to the stoneflies of China (Order Plecoptera). Peking Natural History Bulletin 17: 75–82

[B34] Wu C-F (1962) Results of the Zoologico-Botanical expedition to Southwest China, 1955–1957 (Plecoptera). Acta Entomologica Sinica 11(Supplement): 139–153

[B35] Wu C-F (1973) New species of Chinese stoneflies (Order Plecoptera). Acta Entomologica Sinica 16: 97–118

[B36] Wu C-F Claassen PW. (1934) Aquatic insects of China. Article XXI. New species of Chinese stoneflies. (Order Plecoptera). Peking Natural History Bulletin 9: 111–129

[B37] Yang C-K Yang D (1990) New and little-known species of Plecoptera from Guizhou Province (I). Guizhou Science 8: 1–4

[B38] Yang C-K Yang D (1991) New and little-known species of Plecoptera from Guizhou Province (II). Guizhou Science 9: 48–50

[B39] Yang D Yang C-K (1992) Plecoptera: Perlidae. In: Huang FS (Ed) Insects of Wuling Mountains area, Southwestern China. Science Press, Beijing, 62–64

[B40] Yang D Yang C-K (1993) New and little‐known species of Plecoptera from Guizhou Province (III). Entomotaxonomia 15: 235–238

[B41] Yang D Yang C-K (1995a) Three new species of Plecoptera from Hainan Province. Acta Agriculture Universitatis Pekinensis 21: 223–225

[B42] Yang D Yang C-K (1995b) Plecoptera: Perlidae. In: Wu H (Ed) Insects of Baishanzu Mountain, Eastern China. China Forestry Publishing House, Beijing, 59–60

[B43] Yang D Yang C-K (1996) Four new species of Plecoptera from Nei Mongol. Journal of China Agricultural University 1: 115–118

[B44] Yang D Yang C-K (1998) Plecoptera: Styloperlidae, Perlidae and Leuctridae. In: Wu H (Ed) Insects of Longwangshan. China Forestry Publishing House, Beijing, 40–46

[B45] Ye Y-Zh Qu, W-Y Huang Y-Ch (2002) An overview of Liankangshan Nature Reserve. Science Press, Beijing, 365 pp

[B46] Zwick P (1983) The *Neoperla* of Sumatra and Java (Indonesia) (Plecoptera: Perlidae). Spixiana 6: 167–204

